# Potassium and the kidney: a reciprocal relationship with clinical relevance

**DOI:** 10.1007/s00467-022-05494-5

**Published:** 2022-02-23

**Authors:** Michiel L. A. J. Wieërs, Jaap Mulder, Joris I. Rotmans, Ewout J. Hoorn

**Affiliations:** 1 Department of Internal Medicine, Division of Nephrology and Transplantation, Erasmus Medical Center, University Medical Center Rotterdam, Room Ns403, PO Box 2040, 3000 CA Rotterdam, The Netherlands; 2 Department of Pediatrics, Division of Pediatric Nephrology, Erasmus Medical Center, University Medical Center Rotterdam, Rotterdam, The Netherlands; 3 Department of Pediatrics, Division of Pediatric Nephrology, Leiden University Medical Center, Leiden, The Netherlands; 4 Department of Internal Medicine, Division of Nephrology, Leiden University Medical Center, Leiden, The Netherlands

**Keywords:** Hypokalemic nephropathy, Potassium channel, Potassium supplementation, Salt substitution, Sodium-chloride cotransporter, Tubulopathies

## Abstract

By controlling urinary potassium excretion, the kidneys play a key role in maintaining whole-body potassium homeostasis. Conversely, low urinary potassium excretion (as a proxy for insufficient dietary intake) is increasingly recognized as a risk factor for the progression of kidney disease. Thus, there is a reciprocal relationship between potassium and the kidney: the kidney regulates potassium balance but potassium also affects kidney function. This review explores this relationship by discussing new insights into kidney potassium handling derived from recently characterized tubulopathies and studies on sexual dimorphism. These insights reveal a central but non-exclusive role for the distal convoluted tubule in sensing potassium and subsequently modifying the activity of the sodium-chloride cotransporter. This is another example of reciprocity: activation of the sodium-chloride cotransporter not only reduces distal sodium delivery and therefore potassium secretion but also increases salt sensitivity. This mechanism helps explain the well-known relationship between dietary potassium and blood pressure. Remarkably, in children, blood pressure is related to dietary potassium but not sodium intake. To explore how potassium deficiency can cause kidney injury, we review the mechanisms of hypokalemic nephropathy and discuss if these mechanisms may explain the association between low dietary potassium intake and adverse kidney outcomes. We discuss if potassium should be repleted in patients with kidney disease and what role dietary potassium plays in the risk of hyperkalemia. Supported by data and physiology, we reach the conclusion that we should view potassium not only as a potentially dangerous cation but also as a companion in the battle against kidney disease.

## Introduction

**Fig. 1:**
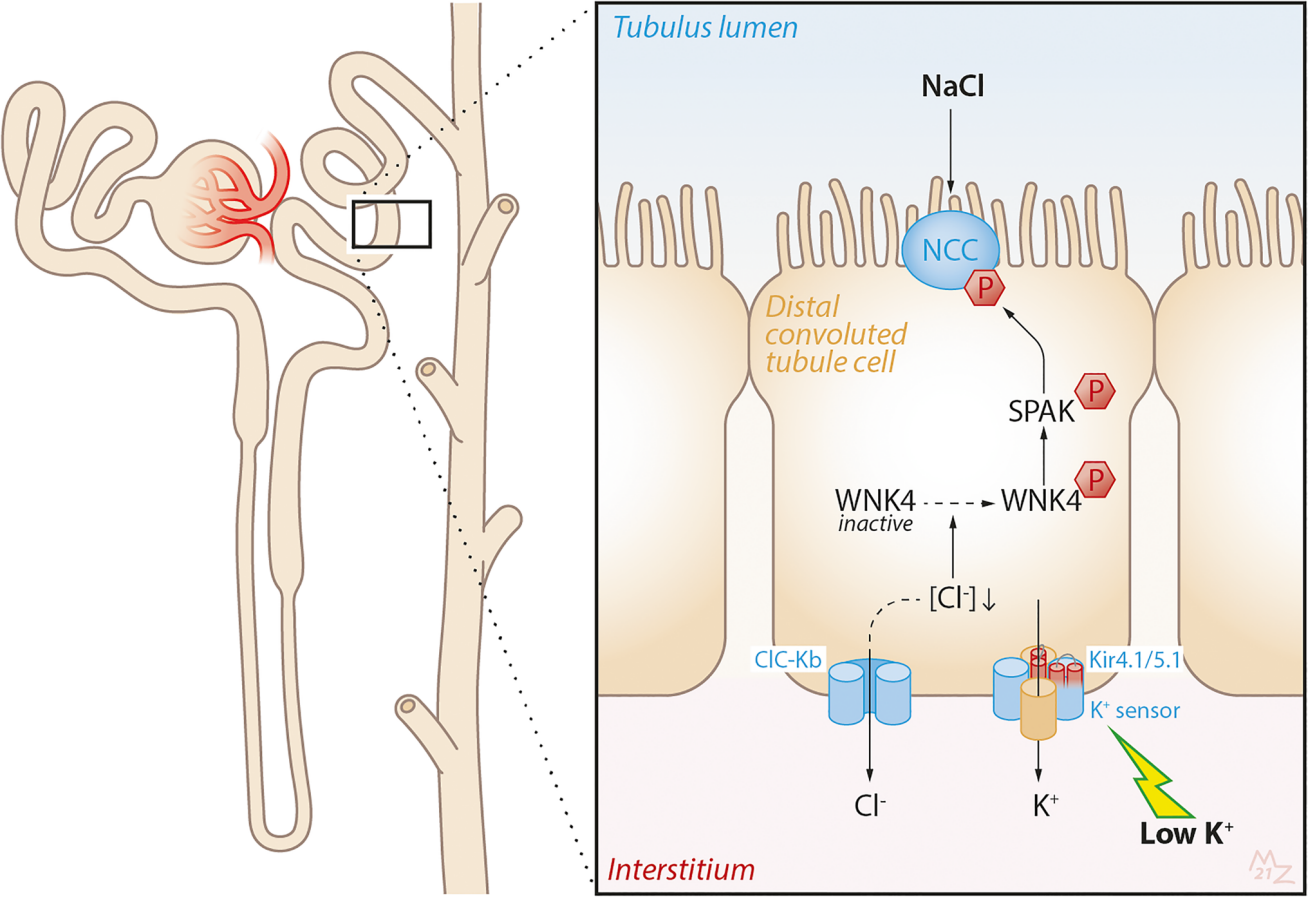
Current model of NCC activation by low K^+^. Low dietary potassium (K^+^) intake and/or a low plasma K^+^ concentration in peritubular capillaries (“low K^+^”) causes hyperpolarization of the basolateral plasma membrane with potassium chloride efflux out of cells. In the kidney, this effect has been most clearly described in the distal convoluted tubule, but probably represents a more general phenomenon [81]. Potassium chloride efflux occurs through the basolateral heteromeric potassium channel Kir4.1/5.1 (encoded by the genes *KCNJ10* and *KCNJ16*) and the chloride channel ClC-Kb (encoded by *CLCNKB*). The ensuing decrease in the intracellular chloride concentration activates the kinases WNK4 and SPAK to phosphorylate and activate the sodium-chloride cotransporter (NCC, encoded by *SLC12A3*). The physiological effect of low K^+^ on NCC is increased reabsorption of sodium chloride by the DCT and a reduction in sodium delivery to downstream nephron segments. This reduces sodium reabsorption by the epithelial sodium channel (ENaC) and electrochemically coupled K^+^ secretion through the renal outer medullary potassium channel (ROMK) (not shown). The “collateral damage” of increased salt reabsorption through NCC is an increase in salt sensitivity

Physicians are trained to deal with disorders of plasma potassium including hypokalemia and hyperkalemia [1, 2]. Disorders of plasma potassium develop when the excretion of potassium (external potassium balance) or the exchange of potassium between the intracellular and extracellular compartments (internal potassium balance) is disturbed [3]. Because the potassium concentration in the extracellular fluid controls the resting membrane potential of excitable cells, disorders of plasma potassium can lead to muscle weakness, paralysis, and cardiac arrhythmias. Several homeostatic hormones such as insulin, catecholamines, and aldosterone maintain extracellular fluid potassium concentration by regulating potassium distribution, excretion, or both [4, 5]. Furthermore, during hypokalemia, skeletal muscle cells release potassium to the extracellular fluid to buffer the fall in potassium concentration [6]. Because potassium primarily resides inside cells, the potassium content in the extracellular fluid volume is only a fraction of total body potassium. Total body potassium is a black box that we tend to ignore clinically. Studies in which total body potassium was measured, however, offer surprising insights. Contrary to what would be expected, patients with kidney failure often have a low total body potassium content [7]. The relevance of this observation is illustrated by the association between total body potassium deficit in patients with kidney failure and increased mortality [8]. Thus, untoward changes in potassium balance can occur without notable changes in plasma potassium or with changes in the normal range. This is also relevant when considering the “evolution” of our diet in terms of electrolytes. Our diet evolved from a high potassium–low sodium diet to a low potassium–high sodium diet. Recommendations for the adequate intake of potassium (dietary reference values) vary per guideline, but generally range between 90 and 100 mmol/day (3,500–4,000 mg/day) for adults and 112 mmol/day (4,400 mg/day) for lactating women [9]. Recommendations for other age groups are 10 mmol/day (400 mg/day) for breastfed infants aged 0–4 months, 15–28 mmol/day (600–1,100 mg/day) for infants aged 4–12 months, and between 20–28 (800–1,100 mg/day) and 90–100 mmol/day (3,500–4,000 mg/day) for children and adolescents [10]. In a worldwide analysis in adults, potassium intake (estimated from urinary excretion) was 2.12 g/day and therefore ~ 40–50% lower than the recommended intake [11]. Patients with chronic kidney disease (CKD) also consume a low potassium diet either habitually or because they were instructed to reduce potassium intake through dietary counseling [12]. Population studies have clearly shown that a low potassium diet is associated with an increased risk of hypertension and cardiovascular morbidity and mortality [13, 14]. Furthermore, several studies have now also shown that a low urinary potassium excretion (as proxy for insufficient dietary intake) is associated with adverse kidney outcomes (reviewed in [15]). Although a potassium-deficient diet can result in overt hypokalemia [16], people consuming a low potassium diet will usually maintain their plasma potassium in the normal range [17]. Therefore, the impact of dietary potassium intake on health is another example of how changes in the external potassium balance can affect the internal potassium balance. The impact of low dietary potassium intake at the cellular level is becoming increasingly clear. For example, at the level of the kidney tubule, a low potassium diet increases the phosphorylation and therefore activity of the sodium-chloride cotransporter (NCC) in the distal convoluted tubule (DCT) (Fig. 1) [18]. This makes sense physiologically, because increased sodium reabsorption by NCC will reduce sodium delivery to downstream potassium-secreting nephron segments and therefore help to conserve potassium [19]. The collateral damage, however, is that NCC-mediated salt reabsorption may result in salt-sensitive hypertension, especially in the setting of a high salt diet [18]. This raises the possibility that the association between low urinary potassium excretion and negative kidney outcomes is mediated by hypertension, although blood-pressure independent effects of potassium deficiency on the kidney have also been demonstrated [20]. The focus of this review will be the reciprocal relationship between potassium and the kidney: the kidney regulates potassium balance, but potassium also affects kidney function. To address this relationship, we will first review how the kidney “senses” potassium and will do so through the insights provided by recently characterized tubulopathies. Subsequently, we will review how a low potassium diet might cause kidney damage by reviewing the clinical and pathophysiological characteristics of hypokalemic nephropathy. Although hypokalemic nephropathy is typically observed in a specific context (e.g., diarrheal diseases, eating disorders, and excessive use of diuretics or laxatives), the mechanisms of hypokalemic nephropathy may also be relevant for the effects of a low potassium diet on the kidney. Recently characterized sex differences in kidney potassium handling will be reviewed to address whether males or females are more susceptible to kaliopenic kidney injury. Next, we will address the effects of low dietary potassium intake in childhood. We will conclude this review with a perspective on current developments regarding the role of dietary potassium in patients with kidney disease.

## New insights from tubulopathies

The effect of low dietary potassium intake on NCC raised the question of how the DCT is capable of sensing potassium. The first clue to the answer was provided by the characterization of a tubulopathy that was coined EAST syndrome (epilepsy, ataxia, sensorineural deafness, tubulopathy) [21]. EAST syndrome is caused by mutations in *KCNJ10*, which encodes Kir4.1, a potassium channel that is expressed in the DCT, brain, and inner ear. EAST syndrome is characterized by a Gitelman-like tubulopathy suggesting that Kir4.1 regulates NCC activity.

This discovery spurred a flurry of research to better understand the signaling pathways involved [22, 23]. Kir4.1 is an important component of basolateral potassium conductance and enables potassium recycling required for sodium–potassium ATPase activity [24]. This has led to the current working model that a decrease in plasma potassium in peritubular capillaries causes potassium efflux through Kir4.1 leading to basolateral membrane hyperpolarization (Fig. 1). The potassium efflux is accompanied by chloride efflux through the chloride channel ClC-Kb. The subsequent reduction in intracellular chloride activates the chloride-sensitive WNK kinases [25, 26]. In turn, the WNK kinases activate SPAK to phosphorylate and therefore activate NCC. In summary, Kir4.1 could be considered a “potassium sensor” (Fig. 1). More recently, another potassium channel has been implicated in NCC regulation. We and others identified that mutations in *KCNJ16* can also cause a Gitelman-like phenotype with sensorineural deafness [27]. *KCNJ16* encodes for Kir5.1, which forms functional heteromers with Kir4.1 (Fig. 1). Indeed, co-expression of mutant Kir5.1 with Kir4.1 in *Xenopus* oocytes significantly reduced electric currents. Remarkably, *KCNJ16* mutations can also cause a different phenotype that is similarly characterized not only by hypokalemia and salt wasting but also by proximal renal tubular acidosis [27]. This phenotypic variability was explained by the fact that Kir5.1 can also interact with Kir4.2 (encoded by *KCNJ15*). Kir4.2 is expressed in the basolateral plasma membrane of proximal tubule cells. Co-expression of these Kir5.1 mutations with Kir4.2 in oocytes also resulted in reduced currents. Depolarization of the basolateral plasma membrane in the proximal tubule could impair bicarbonate exit through the sodium-bicarbonate cotransporter NBCe1 [28]. In patients with these *KCNJ16* mutations, an acid loading test demonstrated a lack of ammonia excretion despite an intact ability to acidify the urine [27]. This suggests that intracellular alkalinization inhibits ammoniagenesis. This also illustrates that basolateral potassium channels play a more general role in the nephron and also regulate acid–base balance [29]. The proposed explanation for the predominant proximal or distal phenotype is the localization of the mutations affecting the potassium channel. *KCNJ16* mutations causing the predominant proximal tubule phenotype were linked to the pore-forming domain near the selectivity filter of the channel, whereas mutations causing the DCT phenotype were located in the N- or C-terminus [27]. This is reminiscent of the phenotypic variability that can be observed with mutations in the basolateral chloride channel ClC-Kb, which may cause a predominant Bartter or Gitelman phenotype [30]. The majority of patients with the *KCNJ16* mutations also had hypomagnesemia. This illustrates the close interaction between potassium and magnesium handling in the kidney. The observation that mutations in *SLC12A3*, *KCNJ10*, and *KCN16* inhibit NCC activity and cause hypomagnesemia illustrates that reduced NCC activity affects magnesium reabsorption. Indeed, both genetic and pharmacological NCC inhibitions reduce the activity of the transient receptor potential melastatin 6 (TRPM6) channel, which mediates transcellular magnesium reabsorption in the DCT. Furthermore, hypomagnesemia can contribute to renal potassium loss either by NCC inhibition (enhancing distal sodium delivery) or reducing the activity of the renal outer medullary potassium channel (ROMK) [31, 32]. Taken together, the regulation of NCC by Kir4.1/5.1 helps to explain NCC activation by low potassium and therefore also why low potassium predisposes to salt-sensitive hypertension (Fig. 1) [33]. However, it does not yet explain how a low potassium diet contributes to kidney injury. Here, it is also relevant to consider other tubulopathies. For example, if chronic hypokalemia would cause kidney injury, one would expect patients with Gitelman syndrome to develop CKD. Similarly, one would also expect patients with distal renal tubular acidosis (dRTA) to be at risk for CKD, especially because metabolic acidosis contributes to kidney injury [34]. Yet, CKD is an uncommon observation in patients with Gitelman syndrome and dRTA. Of note, this could also be related to a lack of longitudinal data, as a recent European survey showed that CKD is more common in patients with dRTA than in the general population, although kidney failure is still rare [35]. Similarly, Gitelman syndrome can cause glomerular proteinuria characterized by focal segmental sclerosis, thickening of the glomerular basement membrane, and podocyte effacement [36]. In contrast to patients with Gitelman syndrome or dRTA, patients with Bartter syndrome more often progress to kidney failure [30]. This discrepancy caused Walsh and colleagues to challenge the concept of “hypokalemic nephropathy” [37]. They showed that, compared to patients with Gitelman syndrome, patients with Bartter syndrome had a lower eGFR, higher plasma potassium, similar blood pressure, and higher plasma renin and aldosterone concentrations. These differences led them to speculate that hypokalemic nephropathy is not caused by chronic hypokalemia itself but rather by a direct effect of aldosterone or volume depletion.

## Hypokalemic nephropathy

**Fig. 2:**
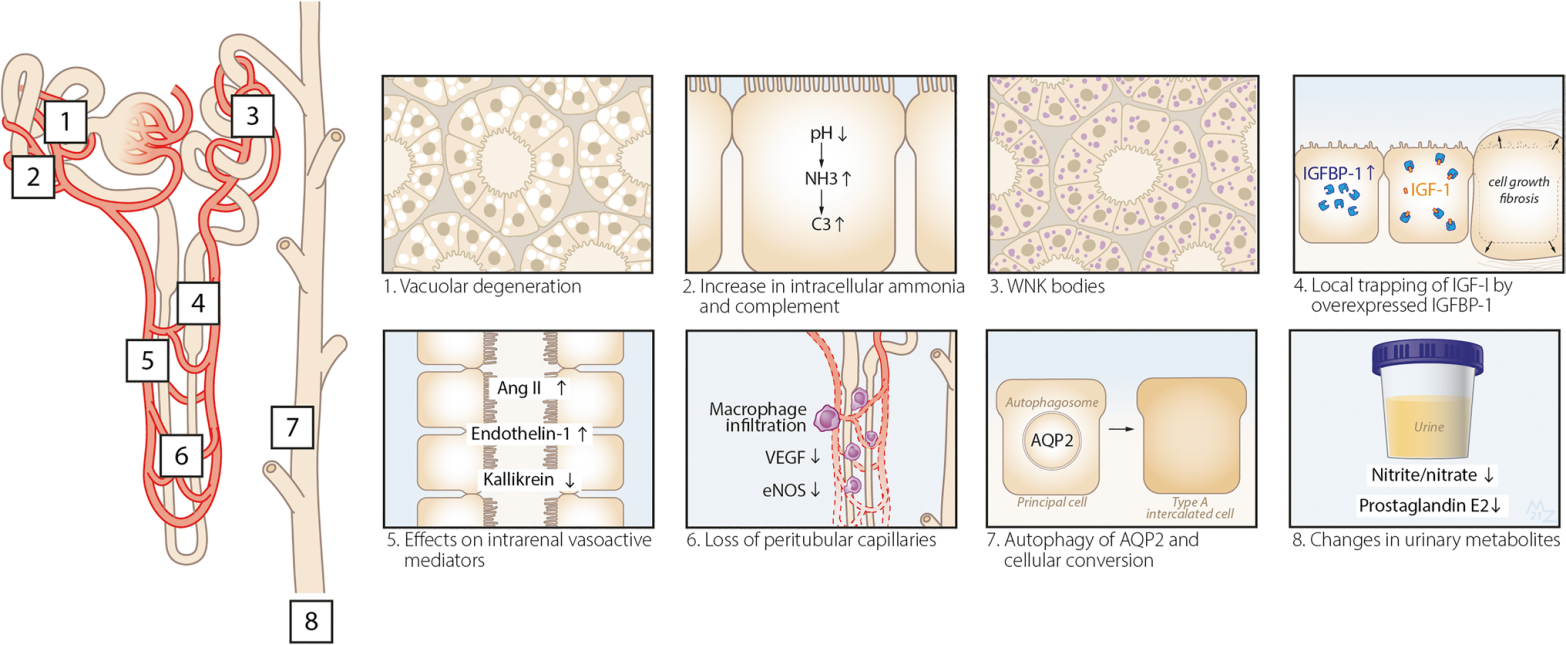
Characteristics of hypokalemic nephropathy. Hypokalemic nephropathy can cause histological or functional changes throughout the nephron. Depicted are the changes reported in previous studies either from kidney biopsies in patients (**panels 1 and** **3)** or observations in animals placed on a potassium-deficient diet (**panels 2, 4–8)**. Hypokalemic nephropathy is characterized by vacuolar degeneration (**panel 1**), which may be observed not only in proximal and distal tubules but also in podocytes and arterial myocytes [39]. More recently, so-called WNK bodies have been described occurring as punctate structures in the distal convoluted tubule (**panel 3) **and consisting of WNKs and SPAK, kinases that are affected by hypokalemia (Fig. 1) [40]. In vivo potassium deficiency has been shown to result in intracellular acidosis and ammoniagenesis with complement activation in cortical tubules (**panel 2)** [41]. The characteristic increase in kidney weight during hypokalemic nephropathy has been explained by local trapping of IGF-I by IGFBP-1, which was mainly observed in the outer medulla (**panel 4**) [42]. Changes in intrarenal and excreted vasoactive peptides and metabolites include an increase in angiotensin II (Ang II) and endothelin-1, and decrease in kallikrein, nitrite/nitrate, and prostaglandin E2 (**panels 5** and **8**) [43]. Hypokalemic nephropathy is also accompanied by loss of peritubular capillaries with macrophage infiltration and a decrease in vascular endothelial growth factor (VEGF) and endothelial nitric oxide synthase (eNOS) (**panel 6**) [47]. Finally, hypokalemic nephropathy causes nephrogenic diabetes insipidus possibly due to autophagy of aquaporin-2 (AQP2) and/or cellular conversion of principal cells to intercalated cells (**panel 7**) [45, 46]

Hypokalemic nephropathy has been reported in patients with malnutrition, eating disorders, vomiting, surreptitious use of laxatives or diuretics, and primary aldosteronism [38]. The common denominators in these disorders are volume depletion and aldosteronism, consistent with the hypothesis of Walsh et al. [37]. Hypokalemic nephropathy often is a progressive form of CKD that can lead to kidney failure. Urinalysis is non-specific. In patients in whom a kidney biopsy is performed, chronic interstitial nephritis and fibrosis are common findings. The microscopic characteristics further include the presence of large and irregular cytoplasmic vacuoles and tubular degeneration (“vacuolar degeneration,” Fig. 2). The vacuoles may be present not only in proximal and distal tubules but also in podocytes and arterial myocytes [39]. More recently, other histological features have been identified in kidney biopsies of patients with hypokalemic nephropathy, which were called “WNK bodies” [40]. WNK bodies are punctate structures in the DCT containing the NCC-regulating WNK and SPAK kinases that localize in non-membrane-bound cytoplasmic regions. Additional characteristics of hypokalemic nephropathy have been delineated in laboratory studies using rats. Compared to rats receiving a potassium-replete diet, a potassium-deficient diet causes hypokalemia, a lower intracellular potassium concentration, lower body weight, and higher kidney weight [41]. In addition to lowering intracellular potassium, potassium deficiency also causes intracellular acidosis. In turn, this results in the production of ammonia, which may function as a local toxin and activate the alternative complement pathway [41] (Fig. 2). A possible explanation of why potassium deficiency impairs body growth but increases kidney growth is the overexpression of insulin-like growth factor-1 binding protein (IGF-1-BP). This may cause local trapping of IGF-1 and therefore low circulating levels of IFG-1 [42] (Fig. 2). Hypokalemic nephropathy was further characterized as an ischemic pattern of injury with intrarenal angiotensin II generation despite suppression of the systemic renin-angiotensin system [43]. This was accompanied by changes in vasoactive mediators, including an increase in endothelin-1 and decreases in kallikrein, nitrite/nitrate, and prostaglandin E2 (Fig. 2). Potassium depletion also causes a rapid downregulation of the water channel aquaporin-2 leading to nephrogenic diabetes insipidus [44]. This may be caused by autophagic degradation of aquaporin-2 [45] or the cellular conversion of principal cells to A-type intercalated cells triggered by suppression of Notch signaling (Fig. 2) [46]. In addition to these tubulointerstitial phenomena, potassium deficiency also impairs kidney angiogenesis. This involves the progressive loss of peritubular capillaries accompanied by macrophage infiltration and loss of vascular endothelial growth factor and endothelial nitric oxide synthase (Fig. 2) [47]. In hypokalemic nephropathy, the normalization of plasma potassium did not reverse salt sensitivity, suggesting a permanent form of kidney injury [43]. Combining the potassium-deficient diet with bicarbonate or endothelin A or B antagonists did prevent the development of kidney injury [41].

## Effects of dietary potassium in children

It is unknown whether low dietary potassium intake in children also leads to kidney injury (Table 1). Experimentally, this has been addressed by placing young rats on a potassium-deficient diet [48]. Potassium-depleted young rats showed a sixfold increase in plasma renin activity which was accompanied by the recruitment of renin-producing cells along the afferent arterioles. Similar to adult rats, the young rats also developed the characteristic features of hypokalemic nephropathy including tubulointerstitial injury with the proliferation of tubular epithelial cells, macrophage infiltration, and fibrosis. Potassium-depleted rats also developed a higher blood pressure which was further characterized by salt sensitivity that persisted after correction of hypokalemia. The relationship between dietary potassium intake and blood pressure has been studied more extensively in children. In a study including 233 children aged 5–17 years, urinary potassium but not sodium excretion was related to systolic blood pressure [49]. These findings were confirmed in a larger cohort with a 10-year follow-up demonstrating that higher potassium intakes were inversely associated with systolic and diastolic blood pressure, whereas sodium intake had no effect on blood pressure [50]. In a placebo-controlled study in 13-year-old children, potassium supplementation for 3 years decreased the blood pressure increase with age in girls but not in boys [51]. Another study applied a dietary approach by randomizing adolescents with prehypertension or hypertension to a “DASH” diet (Dietary Approaches to Stop Hypertension) or routine outpatient nutrition care [52]. The DASH group received more potassium and had a greater decrease in systolic blood pressure *z*-scores than the control group. Importantly, racial differences have been identified in urinary potassium excretion. In one metabolic balance study with fixed intakes, urinary potassium excretion was lower in Black than in White girls possibly due to lower plasma aldosterone [53]. Other proposed explanations are that Black individuals lose more potassium in stool or sweat, have genetic differences in kidney potassium handling, or have lower sodium pump activity [54, 55]. Taken together, the relationship between dietary potassium and blood pressure is already present in childhood, whereas this is less evident for the relationship between dietary sodium and blood pressure. The increasing prevalence of hypertension in children also links to the increasing prevalence of obesity [56–58]. Children with CKD also have a diet that is overly represented by sodium, protein, and calories, but contains too little potassium [59]. This suggests that lifestyle interventions should focus not only on caloric intake but also on sodium and potassium intake. As body weight and urinary sodium and potassium excretion show familial aggregation, such interventions should target the entire family to be successful [60, 61].

**Table 1:** Knowledge gaps

•Is total body potassium decreased in patients with CKD and does this contribute to outcomes?
•Do the mechanisms of hypokalemic nephropathy explain the association between low dietary potassium intake and adverse kidney outcomes?
•Is low dietary potassium intake in children also associated with adverse kidney outcomes?
•Does sexual dimorphism in kidney potassium handling translate to sex differences in the effects of dietary potassium on kidney outcomes?
•Do the positive effects of potassium repletion in patients with CKD outweigh the risk of hyperkalemia?
•Is salt substitution safe and effective in patients with CKD?

## Sex differences

Because most experimental studies on hypokalemic nephropathy were performed in male animals only, it is unclear if there is sexual dimorphism in the development of hypokalemic nephropathy. It is clear, however, that there are sex differences in kidney potassium handling. Female rats and mice have a lower plasma potassium set point which coincides with higher NCC activity, depends on estrogen, and is maintained after a potassium challenge [62]. This lower plasma potassium set point may protect from hyperkalemia during pregnancy when high potassium loads are consumed [62]. Increased NCC activity in female mice is not accompanied by a larger DCT but rather a shorter DCT with higher cellular density of NCC [63]. Female mice also respond more strongly to thiazide diuretics with greater sodium and potassium excretion than males [64]. In female mice, plasma potassium decreases more after exposure to a potassium-deficient diet. This makes female mice more prone to develop nephrogenic diabetes insipidus when reducing dietary potassium [44]. In agreement with these animal data, the prevalence of hypokalemia is higher in females than in males [65]. However, no studies in human subjects have looked into sex differences in hypokalemic complications (Table 1). It is known that females with Gitelman syndrome require significantly more potassium supplementation than males [66]. This sex difference was explained by progesterone which can cause functional aldosterone antagonism with compensatory NCC upregulation. The inability to upregulate NCC in females with Gitelman syndrome could explain the more severe phenotype compared to males. This could also explain why females with Gitelman syndrome typically require even higher doses of potassium supplementation during pregnancy. In contrast, one study found a more severe phenotype in men with Gitelman syndrome [67]. Another study reported same sex siblings with differences in disease severity [68]. Therefore, the role of sex in the disease severity of Gitelman syndrome remains incompletely understood.

## Perspectives for potassium

What should be the next steps to materialize the accumulating evidence for the relationships between dietary potassium intake, blood pressure, cardiovascular, and kidney outcomes? The studies reporting an association between urinary potassium excretion and kidney outcomes in patients with CKD have called for a clinical trial to address causality. The challenge of such a trial was also emphasized as it should strike a balance between investigating the beneficial effects of increasing dietary potassium intake to the dietary reference values without causing excess hyperkalemia (Table 1). In the Netherlands, a placebo-controlled, double-blind randomized clinical trial is currently ongoing to analyze the effects of potassium supplementation (40 mEq/day) for 2 years on kidney function in adult patients with progressive CKD (eGFR between 15 and 45 mL/min/1.73 m^2^) and hypertension [12]. This trial will also be important to analyze the safety of potassium repletion in patients with CKD in terms of hyperkalemia incidence. Here, it is important to consider the effect of dietary potassium on the plasma potassium concentration. In a recent cross-sectional analysis of non-dialysis- and dialysis-dependent patients with CKD, no associations were identified between serum and dietary potassium (assessed by 3-day food records) [69]. We previously did identify a significant but weak correlation between urinary potassium excretion and plasma potassium [12]. Most potassium supplementation studies in subjects with normal kidney function also observed a small but significant rise in plasma potassium of 0.14 mmol/L, which was independent of dose or duration of treatment [70]. The effects of potassium supplementation on plasma potassium will also depend on whether the supplementation is taken with meals and the components of this meal including glucose, sodium, and fiber content [71]. A recent study reported the results of a randomized trial on the effects of a 4-week dietary potassium intervention (40 vs. 100 mmol/day) on serum potassium levels and blood pressure in 29 patients with CKD stage G3 [72]. During the high dietary potassium intake, serum potassium was 0.21 mmol/L higher (leading to hyperkalemia in 2 patients), but failed to reduce ambulatory blood pressure. This is clearly different from potassium supplementation studies in patients with hypertension but without CKD, which show a consistent reduction in blood pressure [73]. The explanation for this discrepancy is unclear, but may relate to the fact that the relationship between urinary potassium excretion and blood pressure follows a U-shaped relationship [74]. CKD-specific factors such as relative hyperaldosteronism, metabolic acidosis, or the uremic milieu may modify or shift this U-shaped relationship. Another possibility is that potassium supplementation should be introduced earlier in the course of kidney disease progression for it to be beneficial. Ideally, a CKD stage is identified with maximal benefit and the lowest risk of hyperkalemia [75]. Longer-term intervention studies in patients with CKD with repeated measurements will be necessary to more definitively assess the effects of adequate potassium intake on plasma potassium, blood pressure, kidney function, and their interdependence. In public health, another potassium-based strategy to positively affect blood pressure is gaining ground, namely salt substitution. Salt substitution refers to the approach to replace ~ 25% of sodium chloride with potassium chloride in discretionary salt use [76]. Two large salt substitution studies in the community were recently completed. The first study (including 2,376 participants) showed that salt substitution reduced the incidence of hypertension by 51% [77]. Of note, in this study, urinary potassium excretion increased, but urinary sodium excretion was unchanged, suggesting that the effect was primarily attributable to increased potassium intake. The second study (including 20,995 participants) observed a similar reduction not only in blood pressure but also in the risk of stroke, major cardiovascular events, and death [78]. In this study, urinary potassium excretion increased and urinary sodium excretion decreased. The effect on cardiovascular outcomes could therefore be attributable to the decrease in sodium intake, the increase in potassium intake, or both. Irrespectively, salt substitution represents a relatively simple intervention that might also benefit patients with CKD. Indeed, modelling studies suggest that the prevention of cardiovascular deaths outweighs excess hyperkalemia deaths, even in patients with CKD [79]. Similar to increasing dietary potassium intake, salt substitution would also be a logical topic for a future clinical trial in patients with CKD. These studies will be necessary to further determine the contribution of dietary potassium to outcomes. In practice, this evidence should be translated to dietary recommendations which fits with the increased focus on plant-based diets in patients with kidney disease [80]. In summary, there is still a lot to learn on the role of potassium in kidney disease (Table 1). Awaiting future studies, it is becoming clear that potassium should not only be considered a potentially lethal cation but may be part of a non-pharmacological approach to improve cardiovascular and kidney outcomes in patients with kidney disease.
